# Prevalence, genetic diversity and eco-epidemiology of pathogenic *Leptospira* species in small mammal communities in urban parks Lyon city, France

**DOI:** 10.1371/journal.pone.0300523

**Published:** 2024-04-10

**Authors:** Marta Garcia-Lopez, Thibaut Lurier, Marie Bouilloud, Julien Pradel, Caroline Tatard, Diana Sepulveda, Gwendoline Anfray, Julie Dussert, Pascale Bourhy, Nathalie Charbonnel, Zouheira Djelouadji

**Affiliations:** 1 USC 1233-RS2GP, VetAgro Sup, University of Lyon, Marcy l’Etoile, France; 2 Biology of Spirochetes Unit, National Reference Center for Leptospirosis, Institut Pasteur, University of Paris Cité, Paris, France; 3 UMR EPIA, INRAE, VetAgro Sup, University of Clermont Auvergne, Saint-Genès-Champanelle, France; 4 UMR EPIA, INRAE, VetAgro Sup, University of Lyon, Marcy l’Etoile, France; 5 CBGP, INRAE, CIRAD, Institut Agro, IRD, University of Montpellier, Montpellier, France; 6 Department of Health, Health and Environment Service, City of Lyon, Lyon, France; 7 Tête d’Or Park Zoo, Lyon, France; 8 Domain of Lacroix-Laval, Marcy l’Etoile, France; National Veterinary Research Institute (NVRI), NIGERIA

## Abstract

Rodents are recognized as the main reservoirs of *Leptospira* spp. Rats, in particular, serve as hosts for the widely predominant *Leptospira interrogans* serovar Icterohaemorrhagiae, found worldwide. Several studies have shown the importance of other reservoirs, such as mice or hedgehogs, which harbor other leptospires’ serovars. Nevertheless, our knowledge of circulating *Leptospira* spp. in reservoirs other than rats remains limited. In this context, we proposed an eco-health approach to assess the health hazard associated with leptospires in urban green spaces, where contacts between human/small mammals and domestic animals are likely. We studied the prevalence, the diversity of circulating strains, and epidemiology of pathogenic *Leptospira* species in small terrestrial mammal communities (rodents and shrews), between 2020–2022, in two parks in Lyon metropolis, France. Our study showed a significant carriage of *Leptospira* spp. in small terrestrial mammals in these parks and unveiled a global prevalence rate of 11.4%. Significant variations of prevalence were observed among the small mammal species (from 0 to 26.1%), with *Rattus norvegicus* exhibiting the highest infection levels (26.1%). We also observed strong spatio-temporal variations in *Leptospira* spp. circulation in its reservoirs. Prevalence seems to be higher in the peri-urban park and in autumn in 2021 and 2022. This is potentially due to differences in landscape, abiotic conditions and small mammal communities’ composition. Our study suggests an important public health relevance of rats and in a lesser extent of other rodents (*Apodemus spp*., *Clethrionomys glareolus* and *Mus musculus*) as reservoirs of *L*. *interrogans*, with rodent species carrying specific serogroups/serovars. We also emphasize the potential hazard associated between the shrew *Crocidura russula* and *L*. *kirschneri*. Altogether, these results improve our knowledge about the prevalence of leptospirosis in an urban environment, which is an essential prerequisite for the implementation of prevention of associated risks.

## 1. Introduction

Leptospirosis is a worldwide bacterial zoonotic disease, caused by the spirochetes of the large diversity pathogenic *Leptospira* ssp. It is considered by the World Health Organization (WHO) as a public health problem [1, 2]. The infection is estimated to cause one million human cases and around 60,000 deaths annually [3]. Clinical manifestations of leptospirosis range from mild febrile illness to life-threatening renal failure, pulmonary hemorrhage, and/or cardiac complication [4].

Leptospires are maintained in several wild and domestic animal hosts through the renal carriage and are excreted in the urine for several months [5]. Infection occurs through direct contact with the urine of infected animals, or most often indirectly by exposure to leptospires contaminated environment [5, 6]. Rodents, in particular rats, are considered the most important reservoir of pathogenic *Leptospira* spp. due to their large number and their proximity with humans and domestic animals. Therefore, they play a major role in the epidemiology of leptospirosis [7, 8]. However, different studies reported the presence of additional animal reservoirs other than rats, that harbor different *Leptospira* species and serovars, including strains with different virulence for humans and animals [9–12].

Leptospirosis is associated with various risk factors such as global warming which favors the survival of the bacteria. Heavy rainfall or flooding can lead to outbreaks of leptospirosis by bringing pathogens to the surface [8, 12, 13]. Lastly, human exposure to contaminated environment is associated with recreational activities [4, 14]. These recent years, many cases have been reported in urban areas [15]. In cities, the risk of leptospirosis is increasing due to several factors like urbanization and infrastructure, which expand and encroach on natural habitats, altering the structure and function of the landscape, and resulting in increased interactions between humans and wildlife [16, 17]. These changes are accompanied by a marked reduction in biodiversity in cities. On one hand, many species may disappear entirely from the urban environment. On the other hand, the urbanization process may favor a few wild animal species; including rats and mice, that have a strong ability to adapt to these environments [18]. Dampened competition and illimited access to food may result in unusually high population densities. These latter, coupled with inadequate waste management and poor sanitation practices can result in a potential risk for the (re)emergence of zoonotic infectious diseases like leptospirosis [19–21]. Moreover, the prevalence and distribution of circulating leptospires strains in urban areas strongly depends on the species of local fauna present and their interactions [22].

In metropolitan France, the incidence of human leptospirosis is among the highest in Europe with 800 cases per year and an incidence of one case per 100,000 inhabitants [14, 23, 24]. While the source of human infection in metropolitan France remains unclear [14, 24], exposure to rodent reservoirs is reported as an important risk factor for leptospirosis [3, 15, 25]. Lyon is the third biggest city in France, a city which has been expanding over time. Studies conducted in Lyon between 2014 and 2015 among rat populations have shown a high prevalence of 26% [19]. This prevalence was higher in neighborhoods with high human density and low income. In addition, the strains identified in rats from Lyon (*L*. *interrogans* serovars Copenhageni and Icterohaemorrhagiae) were found to be identical to the reference strains isolated from humans [26]. However, there is a lack of data regarding the prevalence, the diversity and types of circulating strains among other reservoirs like small terrestrial mammals, the seasonal impact, and the diversity of carrier species in urban areas. Understanding the influence of these factors on the circulation and maintenance of *Leptospira* spp. in wildlife has never been studied in the geographical context of Lyon city. Such knowledge would be invaluable in designing suitable preventive measures for both humans and domestic animals on a local level within urban areas.

In this context, we have developed an eco-health approach to assess the health hazards associated with leptospires in Lyon. We focused on urban green spaces, where contact between human/small mammals and domestic animals is likely because they share the same environment and the risk of zoonoses may be high [27]. The objectives of this study were i) to evaluate the prevalence of pathogenic *Leptospira* spp. in small terrestrial mammals present in two urban and peri-urban parks in Lyon metropolis, France, ii) to investigate the biotic and abiotic factors that may shape the variability of the prevalence levels and iii) to identify *Leptospira* strains circulating in these small mammal communities using molecular methods.

## 2. Material and methods

### 2.1. Ethics statement

This study was part of the European Biodiversa BioRodDis project (https://www6.inrae.fr/biodiversa-bioroddis). The CBGP laboratory has approval (E-34-169-001) from the Departmental Direction of Population Protection (DDPP, Hérault, France) for the sampling of small mammals and the storage and use of their tissues. All procedures related to small mammals captured in this study complied with the ethical standards of the relevant national and European regulations on the protection of animals used for scientific purposes (Directive 2010/63/EC revising Directive 86/609/EEC, adopted on 22 September 2010). Briefly, rodents were captured alive and sacrificed by cervical dislocation, after sedation (using isofluorane) for rodents weighing more than 150 g. To minimize stress and suffering, we used traps equipped with plastic boxes filled with cotton and bait. Additionally, traps were inspected each morning. These procedures have undergone validation by the regional ethics committee “Comite d’Ethique pour l’Expérimentation Animale Languedoc Roussillon n°36”in 2020.

### 2.2. Study areas and sample collection

Small terrestrial mammals (*Rodentia* and *Soricomorpha*) were captured during spring and autumn between 2020 and 2022 in Lyon metropolis, France, within two sites: an urban park (FRPLTO: Tête d’Or park) and a peri-urban park (FRPDLL: Domain of Lacroix-Laval). The urban park studied is the largest one in France, with 117 hectares. It is located in central Lyon city, and includes a zoo, a botanical garden, a lake, buildings, restaurants, landfills, playground for kids, and large green esplanades. The peri-urban park is located within the city of Marcy-l’Étoile, 15 km away from Lyon. It includes 115 hectares of meadows, ponds, rivers and woods. It also houses a castle and a restaurant.

All information about trapping and dissection methods of small mammals are detailed in Pradel et al. [28]. Briefly, the animals were trapped alive in two different ways: either from lines of 20 traps set for the capture of small mammals (11 lines in the urban park and 10 lines in the peri-urban park), or from metal mesh traps set opportunistically for rats. The location of the traps remained the same throughout the study, which consisted of 5 trapping sessions (3 springs and 2 autumns). Small mammal species were identified using morphological criteria in the field and molecular methods, when necessary, as described by Pradel et al. [28]. Animals were euthanized and dissected aseptically, according to the protocols described in Herbreteau et al. [29]. Animals were weighed and morphological measurements were conducted, including body length and tail length. Sex was recorded and sexual maturity was inferred considering testes length and position of testicles (abdominal or descended into the scrotal sac) and seminal vesicle development (visible or not present) for males or vaginal opening, nipples (visible or inappreciable), gestation and uterus size for females [29].

### 2.3. DNA extraction and *Leptospira* spp. detection by Real-time PCR

#### DNA extraction

Kidney tissues were collected in sterile tubes with ethanol and stored at 4°C before analyses. DNA was isolated by cutting a piece of tissue at the cortico-medullary junction (<30 mg) from the kidney tissue sample. Total DNA was extracted from kidneys using the BioBasic EZ-10 96 well plate genomic DNA isolation kit for animals (Euromedex, Souffelweyersheim, France) following the manufacturer’s instructions. DNA was quantified using Nanodrop technology (Thermo Scientific, Lissieu, France).

#### RT-PCR-screening by *lipL32* gene

Real-time PCR (RT-PCR), targeting a partial region specific to pathogenic *Leptospira* spp. of the gene encoding the 32-kDa lipoprotein (*lipL32*), was performed immediately after extraction following the protocol described in Dobigny et al. [30, 31]. RT-PCR reactions were performed using a TaqMan probe and FastStart Taq (Roche Diagnostics, Meylan, France) on the LC480 LightCycler (Roche Diagnostics, Meylan, France) in 384-well plates. We included 2 μL of DNA in 10 μl final volume for each reaction. All samples were duplicated and positive/negative controls were included in each plate. Samples with a Cycle threshold (Ct) higher than 40 were considered as negative.

#### RT-PCR-screening by 16S rRNA (*rss* gene) pathogenic *Leptospira* spp. Gene

In parallel, a RT-PCR targeting a partial region of the pathogenic *Leptospira* spp. 16S rRNA *(rrs* gene) [32] was performed. This second RT-PCR was used to increase the percentage of positive samples. In 2020, the 16S rRNA RT-PCR was performed on DNA extract used for the *lipL32* analysis. However, the correct conservation of these DNA was not guaranteed due to the transport condition. Then, from 2021–2022, we minimized DNA degradation problems associated with frozen storage, and DNA was again extracted from fresh kidneys (>25 mg) using Qiagen DNeasy® Blood & Tissue Kit (QIAGEN, Courtaboeuf, France), following the manufacturer’s instructions. The RT-PCRs were performed within less than 24 hours following DNA extraction using the AgPath-IDTM One-step RT-PCR Reagents (Life technologies, Lissieu, France), according to the manufacturer’s instructions. Each reaction contained 19 μL of mix and 6 μL of extracted DNA. Positive and negative controls consisting of 6 μL of *L*. *interrogans* serovar Copenhageni CG6 DNA and free water were included, respectively. Samples with a Ct higher than 40 were considered negative. Beta-actin gene was used as an internal positive control to confirm the removal of PCR inhibitors in the samples and the validation of *Leptospira* spp. amplification.

### 2.4. Statistical analyses

All statistical analyses were performed using R software [33]. The 95% confidence intervals for prevalence were calculated by Pearson’s chi-squared using the conf.int function, selection option (prop.test). The objective was to identify the factors that influenced *Leptospira* spp. presence in small mammals’ kidney using a generalized linear mixed model (GLMM) [34], with the glmer function of the *lme4* package [35]. The predicted variable was the presence / absence of *Leptospira* spp. DNA detected by RT-PCR. A sample was considered positive if at least one of the two approaches described above (16S rRNA or *lipL32* RT-PCR) provided a positive result (Ct ≤ 40). It was considered negative when both 16S rRNA and *lipL32* RT-PCR approaches provided negative results (Ct > 40). The factor trap-position was included as a random effect to account for the location of small mammals captured. Individual demographic variables (species, sex, weight as a proxy of age and sexual maturity) as well as abiotic factors (site, season and year) were included as fixed effects in the complete model. To account for interspecies variability, the weight of each small mammal was divided by the mean weight of the animals captured in the corresponding species.

Variable selection was performed using the *MuMIn* (Multi-Model Inference) package [36] in R with the automated model selection option (*dredge*) to calculate the corrected second-order Akaike information criterion (AIC) for all possible sub-models. Best-fitting models were defined as those that were within a ΔAIC of <2 of the best model (lowest AIC). Post-hoc Tukey comparisons between each pair of significant factors with more than two modalities were performed with the glht function of the *multcomp* package [37].

Correct fit of the logistic regression was assessed using Pearson residual analysis. All parameters were null-checked using a Wald test and considered significant at a p value <0.05.

To assess the risk of bias associated with the inclusion of 16S rRNA PCR results obtained in 2020 with a different extraction process, the final model was re-run by excluding the 2020 data. We checked whether the risk factors identified from the full dataset were preserved in this data subset.

### 2.5. *Leptospira* spp. genotyping analyses

Genotyping was performed using the amplification and sequencing of *lfb1*-gene on positive samples, using primers described by Mérien et al. [38]. PCRs were performed using the HotStarTaq DNA Polymerase kit (QIAGEN, Courtaboeuf, France) according to the manufacturer’s instructions. All positive products were Sanger sequenced by the GenoScreen sequencing platform (Lille, France). Sequence analyses and phylogenetic tree were made using the software BioNumerics V7.6 (Applied-Maths), as described by Garcia-Lopez et al. [24]. *Leptospira* species were identified using sequence information from the BIGSdb-Pasteur database (https://bigsdb.pasteur.fr/leptospira/).

**Fig 1:**
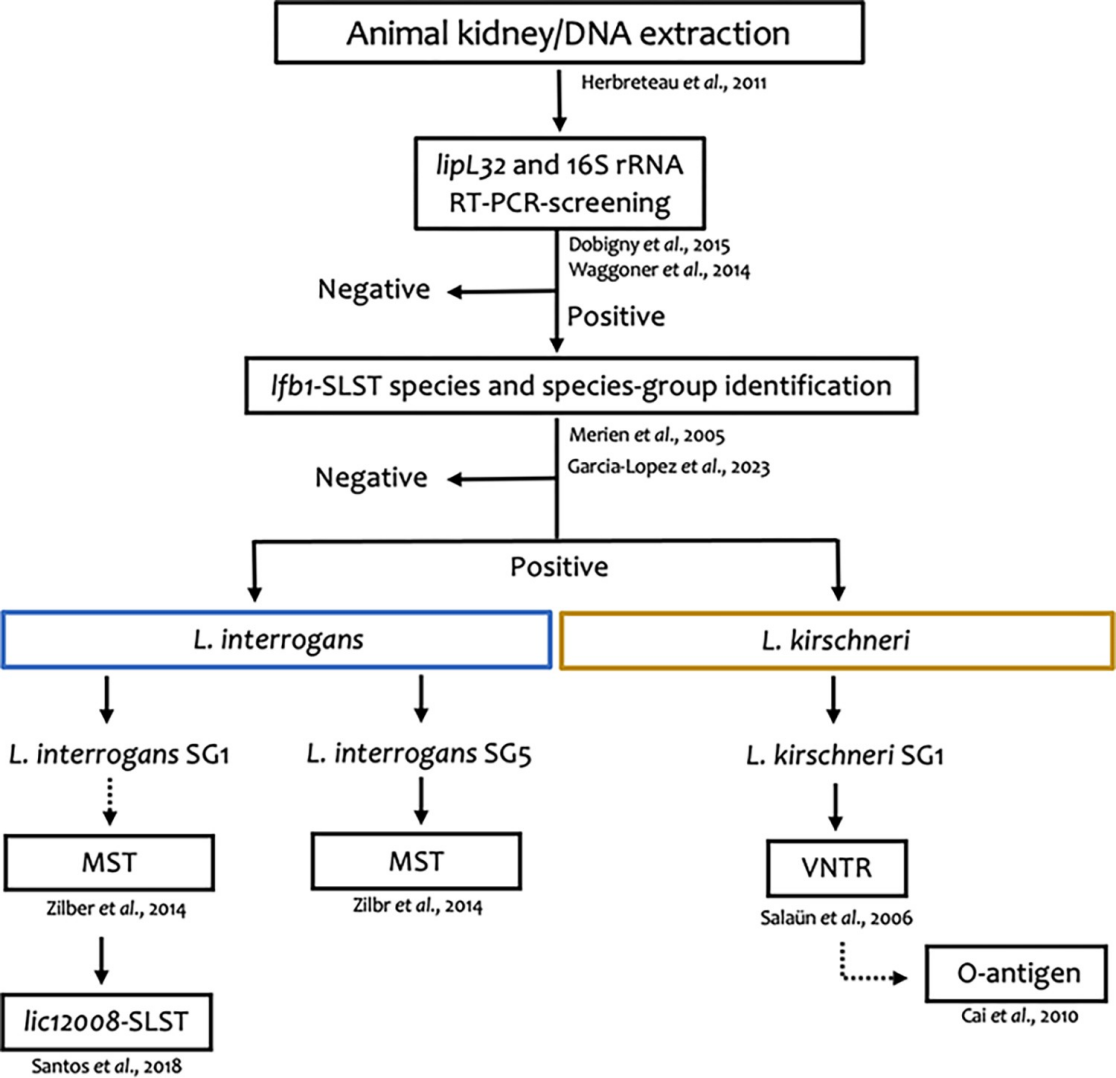
Flowchart of the molecular analyses performed to discriminate *Leptospira* species, serogroup and serovar. SLST: single-locus sequence typing; VNTR: Variable Number Tandem Repeat; MST: Multi Spacer Typing. Dotted arrows indicate verification tests.

After this first level of *lfb1*-gene identification, all positive samples were genotyped following different methods as described in **Fig 1** [39–42], using HotStarTaq DNA Polymerase kit (Qiagen) according to the manufacturer’s instructions. Positive samples identified as *L*. *interrogans* specie were screened using Multispacer sequence typing (MST) as described by Zilber et al. [41] based on the amplification and sequencing of three intergenic regions, MST1, MST3, and MST9. The sequences obtained were compared to the MST database available on Genbank [41]. Positive samples belonging to the *L*. *interrogans* serogroup Icterohaemorrhagiae were then genotyped using the *lic12008* single-locus sequence typing (SLST) described by Santos et al. [42]. This analysis enabled the distinction between the serovars Copenhageni and Icterohaemorrhagiae. The *lic12008* sequences were compared to the database published by Santos et al. [42].

Variable Number Tandem Repeat (VNTR) was performed on positive samples identified as *L*. *kirschneri* species, as described by Salaün et al. [39]. The VNTR profiles obtained were compared to the framework previously published [39]. Finally, O-antigen typing was performed on all VNTR positive samples identified as belonging to the serogroup Grippotyphosa using primers described by Cai et al. [40] for confirmation. O-antigen amplified products were Sanger sequenced and the sequences obtained were analyzed and compared to the database described by Cai et al. [40].

## 3. Results

### 3.1. Description of the small mammals analyzed

A total of 595 animals were analyzed in this study. Among them, 512 rodents belonged to four murine species of the *Muridae* family [*Apodemus sylvaticus* (n  =  255), *Apodemus flavicollis* (n  =  38), *Rattus norvegicus* (n  =  69) and *Mus musculus* (n  =  44)] and two vole species of the *Cricetidae* family [*Clethrionomys* (syn. *Myodes) glareolus* (n  =  77) and *Microtus arvalis* (n  =  29)]. In addition, 83 shrews belonging to one species of the family *Soricidae* [*Crocidura russula*] were also captured and analyzed (**Table 1**).

**Table 1:** Number of animals analyzed for each park as a function of year, season, sex, sexual maturity and species.

Characteristics		Total	Site	
			Urban Park FRPLTO	Peri-urban Park FRPDLL
**n**		595	355	240
**Year**	**2020**	156	115	41
	**2021**	369	181	188
	**2022**	70	59	11
**Season** (all years included)	**Spring**	284	178	106
	**Fall**	311	177	134
**Sex** (all years included)	**Male**	332	211	121
	**Female**	263	144	119
**Sexual maturity** (all years included)	**Immature**	153	87	66
	**Mature**	349	207	142
	**Unknown**	93	61	32
**Species** (all years included and by phylogenetic order)	***Rattus norvegicus***	69	69	0
	***Mus musculus***	44	43	1
	***Apodemus sylvaticus***	255	147	108
	***Apodemus flavicollis***	38	0	38
	***Clethrionomys glareolus***	77	0	77
	***Microtus arvalis***	29	28	1
	***Crocidura russula***	83	68	15

**Fig 2:**
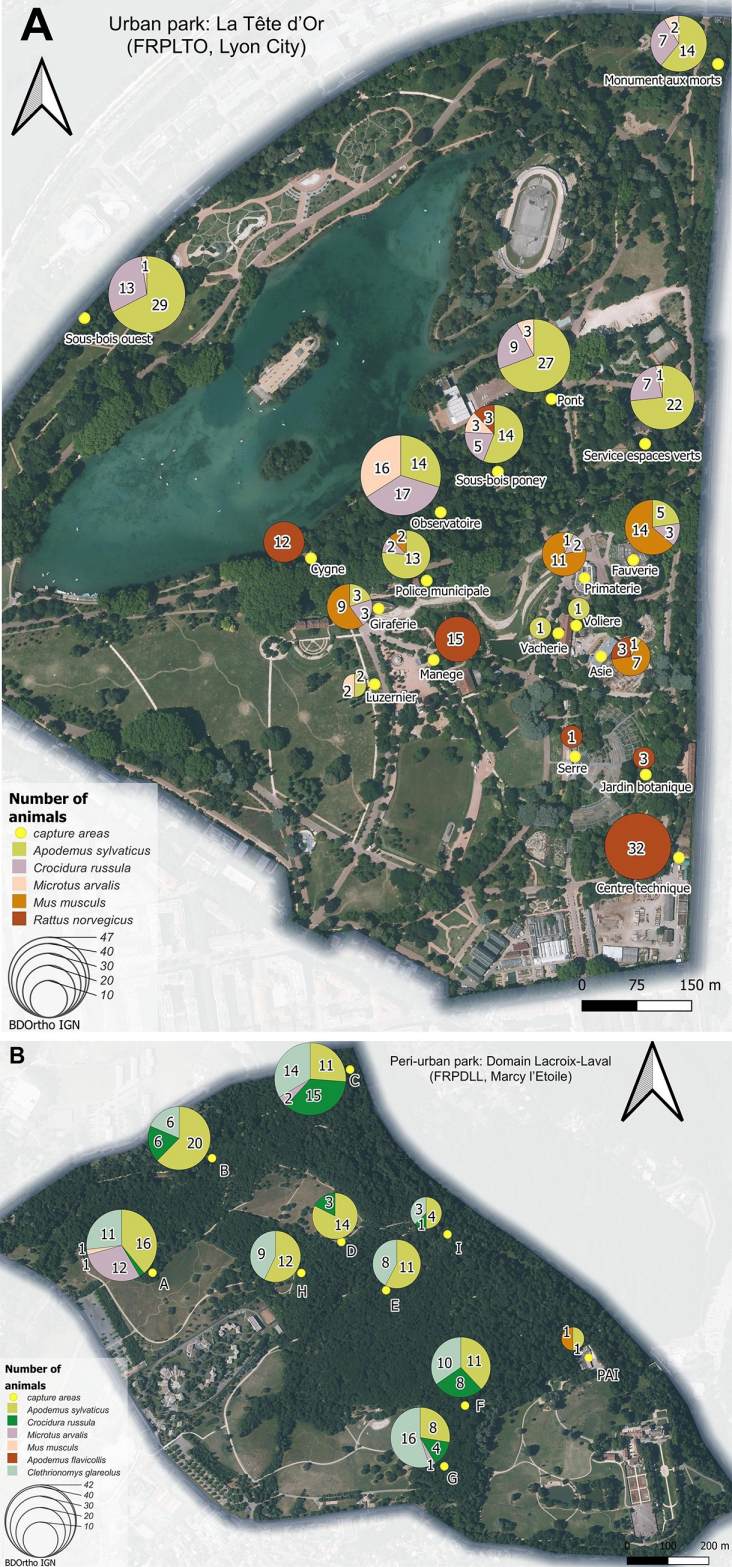
Spatial distribution of the small terrestrial mammals analyzed from A) the urban park La Tête d’Or (FRPLTO, Lyon City) and B) the peri-urban park Domain Lacroix-Laval (FRPDLL, Marcy l’Etoile). Pictures were extracted from ®IGN ®BD ORTHO (2023), that is from a governmental openly available source. Yellow dots correspond to trapping areas. Colors within circles symbolize different species, and the size of each circle is proportional to the total number of animals analyzed per trap line.

The distribution of individuals captured in each park is presented in **Fig 2.**

### 3.2. *Leptospira* spp. screening

Out of the 595 animals analyzed, 68 were positive, among which 19 positive samples were detected by both techniques (*lipL32* RT-PCR and 16S rRNA RT-PCR), 12 positive samples were detected only by *lipL32* RT-PCR and 37 positive samples only by 16S rRNA RT-PCR. Detailed results are presented in **S1 and S2 Tables**. All positive samples were considered in further analyses to increase the sensitivity of *Leptospira* spp. detection.

Overall, the global prevalence reached 11.4% [95%CI = 9.0–14.3]. It varied from 0 to 26.1% in the different small mammal species. *Leptospira* spp. were detected in all small mammal species, except *Microtus arvalis*, where no individuals tested positive. Moreover, *Leptospira* spp. prevalence varied between sampling sessions, as presented in **Table 2**.

**Table 2:** Observed levels of pathogenic *Leptospira* spp. prevalence and 95% confidence intervals of pathogenic *Leptospira* spp. among small mammal species per sampling session.

Common name (Scientific name)	*Leptospira* spp. Number of positive animals/total number of animals tested Prevalence 95% confidence interval					
**Sampling session**	Spring 2020	Autumn 2020	Spring 2021	Autumn 2021	Spring 2022	All sessions *n =* 595
**Brown rat** **(*Rattus*** ***norvegicus*)**	0/0	0/8	4/15 26.7% 8.9–55.2%	7/23 30.4% 14.1–53.0%	7/23 30.4% 14.1–53.0%	**18/69** **26.1%** **16.6–38.3%**
**House mouse** **(*Mus musculus*)**	0/8	0/10	0/9	2/11 18.2% 3.2–52.2%	1/6 16.7% 0.9-63-5%	**3/44** **6.8%** **1.8–19.7%**
**Wood mouse** **(*Apodemus sylvaticus*)**	0/25	2/37 5.4% 0.9–19.5%	9/79 11.4% 5.7–21.0%	14/88 15.9% 9.3–25.6%	3/26 11.5% 3.0–31.3%	**28/255** **10.9%** **7.5–15.6%**
**Yellow-necked mouse** **(*Apodemus flavicollis*)**	0/0	2/11 18.2% 3.2–52.2%	1/15 6.7% 0.35–33.9%	4/11 36.4% 12.4–68.4%	0/1	**7/38** **18.4%** **8.3–34.9%**
**Bank vole** **(*Clethrionomys*** ***glareolus*)**	0/0	0/11	1/34 2.9% 0.15–17.1%	2/26 7.7% 1.3–26.6%	1/6 16.7% 0.9–63.5%	**4/77** **5.2%** **1.7–13.5%**
**Common vole** **(*Microtus arvalis*)**	0/14	0/0	0/2	0/9	0/4	0/29
**Greater white-toothed shrew** **(*Crocidura*** ***russula*)**	0/0	1/32 3.1% 0.16–17.9%	0/13	5/34 14.7% 5.5–31.8%	2/4 50% 15.0–84.9%	**8/83** **9.6%,** **4.6–18.6%**

### 3.3. Abiotic and biotic factors associated with *Leptospira* spp. carriage

The best model explaining *Leptospira* spp. prevalence (AICc = 344.4) included the factors site, small mammal species, year and season. It explained 23% of the total variance (**S3A Table)**.

**Fig 3:**
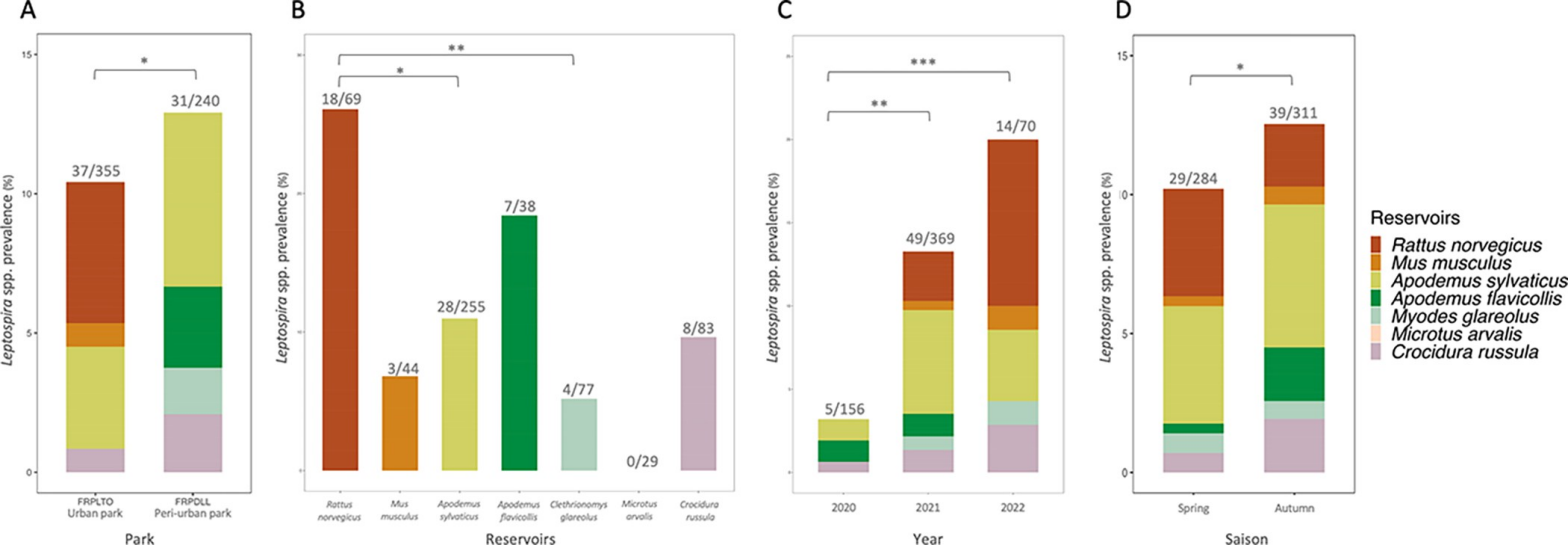
Variations in the levels of *Leptospira* spp. prevalence between parks (A), small mammal species (B), year (C), and season (D). *, ** and *** correspond to p value higher than 0.05, 0.01, 0.001 from the GLMM or from the post-hoc multiple tests (Tukey comparisons for species and year).

The probability of being infected with *Leptospira* spp. was significantly lower in the urban park (FRPLTO: 10.4%) compared to the peri-urban one (FRPDLL: 12.9%). Considering that all other risk factors were equal (year, season and small mammal species), the probability of finding an infected small mammal in the peri-urban park was twofold higher than in the urban park (logistic regression, p = 0.038, OR = 2.21, 95%CI = 1.04–4.66) (**Fig 3A**).

We observed significant differences in *Leptospira* spp. prevalence between small mammal species. Post-hoc tests revealed that the levels of prevalence in *Rattus norvegicus* were significantly higher than those observed for *Apodemus sylvaticus* (p value = 0.039) and *Clethrionomys glareolus* (p value = 0.005) (**Fig 3B**).

We found significant temporal variations of *Leptospira* spp. prevalence with the highest levels observed in 2022 (20%, 95%CI = 11.7–31.6) compared to 2021 (13.3%, 95%CI = 10.1–17.3) and 2020 (3.2%, 95%CI = 1.19–7.7). The prevalence was more than five times (respectively twelve times) higher in 2021 (logistic regression, p = 0.0016, OR = 4.81, 95%CI = 1.81–12.74; respectively 2022 (logistic regression, p = 0.0001, OR = 12.45, 95%CI = 3.47–44.67) compared to 2020 (**Fig 3C**). Moreover, significantly more *Leptospira*-positive small mammals were found in autumn than in spring (logistic regression, p = 0.0163, OR = 2.24, 95%CI = 1.16–4.31), where 10.2% of the small mammals captured were positive in spring (29/284) and 12.5% in autumn (39/311) (**Fig 3D**).

Other individual characteristics (sex, sexual maturity and weight) did not significantly explain small mammals’ infection by *Leptospira* spp. The mixed logistic regression analysis performed after excluding animals trapped in 2020 provided results that were similar to those obtained with the whole dataset (**S3B** Table). All the estimated values were of the same sign and of the same order of magnitude, but with wider confidence intervals and certain p values that became greater than 0.05 (for example location and year).

### 3.4. *Leptospira* spp. genotyping analyses

Due to the low concentration of *Leptospira* spp. DNA in small mammals’ kidneys, the successful identification of *Leptospira* species-group using *lfb1*-SLST was achieved in only 27 out of the 68 positive samples (39.70%). These later had cycle thresholds (Ct) ranging between 16.44 and 36.79. In 95% of the samples with Ct >37 (41/68 samples), genotyping was not successful.

**Fig 4:**
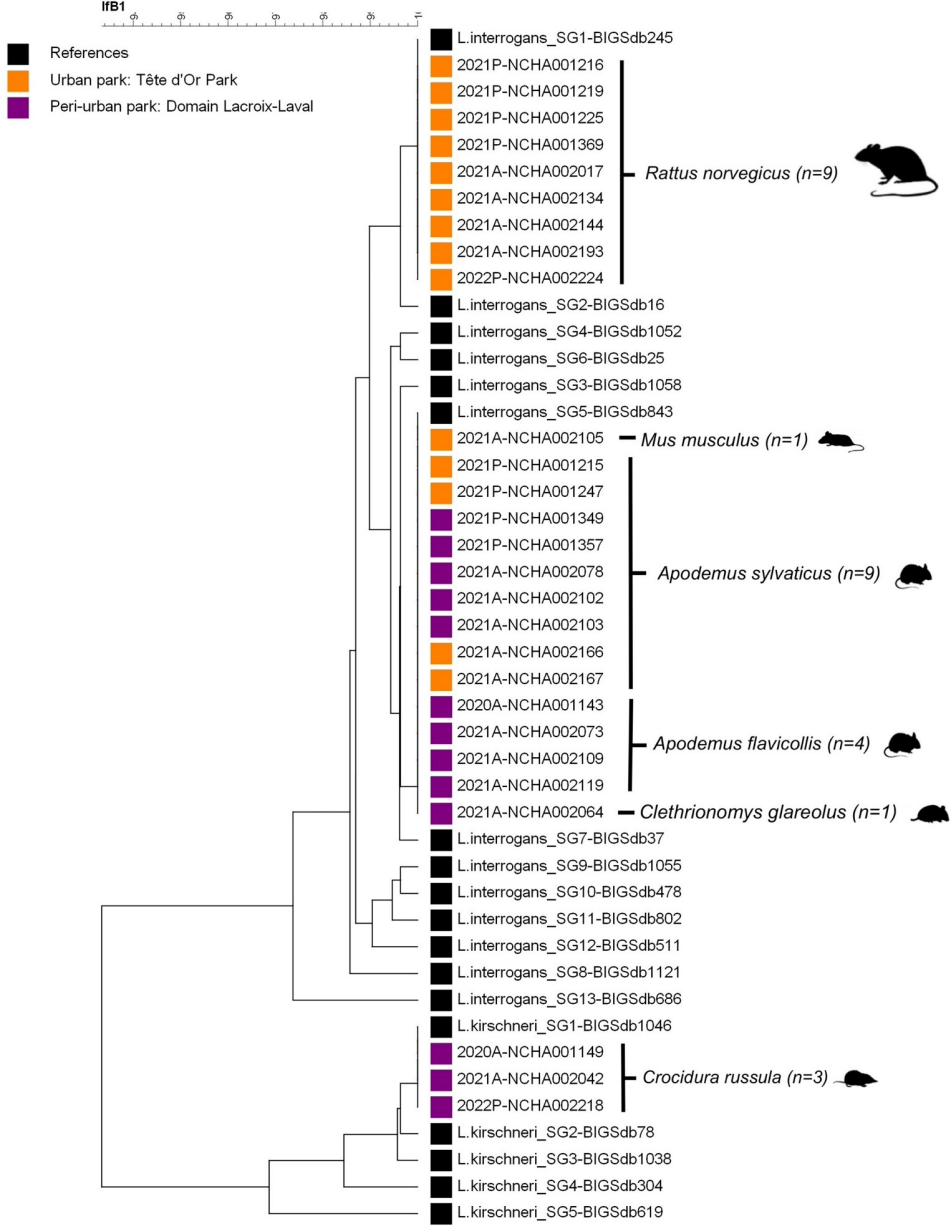
Phylogenetic tree inferred from *Leptospira* spp. detected in the two urban parks, based on *lfb1* partial gene sequences (334 bp) originating from animal sample specimens and reference strains [24]. Reference strains were selected from the BIGSdb database and their BIGSdb accession numbers are indicated. *L*. *interrogans* SG1 corresponding to serovar Icterohaemorrhagiae/copenhagageni, *L*. *interrogans* SG5 serovar Bratislava/Lora/Jalna/Muenchen/Fugis/Bataviae/Valbuzzi and *L*. *kirchneri* SG1 corresponding to serovar Grippotyphosa/Valbuzzi/Vanderhoedeni/Pomona/Mozdok/Bim/Mwogolo/Sokoine [24].

Sequence analyses based on *lfb1* were performed on 27 samples (**Fig 4**), revealing the presence of two *Leptospira* species, namely *L*. *interrogans* SG1, SG5 (88.90%, n = 24) and *L*. *kirschneri* SG1 (11.10%, n = 3) [24]. No co-infection with these two *Leptospira* species was detected among the samples sequenced.

All rodents were infected with the same species, *L*. *interrogans*. The MST profiles was successfully determined for 16/24 samples only. They revealed the presence of two genotypes. The *L*. *interrogans* genotype related to the Icterohaemorrhagiae serogroup was identified in rats (n = 5). The *L*. *interrogans* genotype related to the Australis serogroup was identified in wood mice (n = 6), yellow-necked mice (n = 4), and bank voles (n = 1). For six animals, including three *A*. *sylvaticus*, two *R*. *norvegicus*, and one *M*. *musculus*, MST profiles were incomplete (**S4A Table)**.

*L*. *kirschneri* was detected in three samples, all of them corresponding to the greater white-toothed shrew *C*. *russula*. The three VNTR markers used (VNTR-4, VNTR-7, and VNTR-10) allowed the identification of the 0-2-9 VNTR profile, which corresponded to the *L*. *kirschneri* Grippotyphosa serogroup and Valbuzzi serovar. The O-antigen typing confirmed the Grippotyphosa serogroup identification (**S4B Table)**.

## 4. Discussion

This study represents the first investigation of the prevalence of *Leptospira* spp. in small mammals, encompassing various species found in two urban and peri-urban parks in Lyon. It takes into account the geographic, seasonal, and habitat-related factors that were likely to influence *Leptospira* spp. prevalence over a three-year period. Using molecular methods, our study highlights the presence and genetic diversity of leptospires in six out of the seven species of small mammals inhabiting the parks, elucidating their potential contribution to environmental contamination.

This study demonstrates an average *Leptospira* spp. prevalence of 11.4% among the 595 small territorial mammals analyzed. The prevalence observed here corroborates the results of a previous study conducted in Corse, France (10.4%) [11]. Nevertheless, higher levels of *Leptospira* spp. prevalence were detected in nearby European countries with similar climate like Germany (20.7%) [43] and Italy (18.9%) [44]. The reported differences in prevalence can be attributed to several factors, including methodological (the target PCR method and its sensitivity, the presence of samples with low-level positivity) and biological ones (the trapping locations, the species of animals sampled).

In this study, two genes (*rrs* gene - 16S rRNA and *lipL32* gene) were targeted with quantitative PCR to enhance the detection sensitivity of *Leptospira* spp. in samples collected from small mammals. Our results confirm the higher sensitivity of 16S rRNA and/or the higher specificity of *lipL32*, as previously reported in other studies [45]. The 16S rRNA target showed a 36.7% improvement in the detection of low-level positive samples, especially for detecting a higher number of positive animals with Ct values above 30. This method should therefore be favored in the future [46, 47]. At the opposite, several studies affirm that *lipL32* has better specificity to detect all pathogenic *Leptospira* spp., because the *lipL32* gene is present only in pathogenic species [48]. As such, this option is the most widely used among studies aiming at detecting leptospires in humans, animals and the environment [11, 49, 50]. However, there is no obvious reason explaining why 12 samples were positive to *lipL32* only (S2 Table). Then, to mitigate the risk of missing potential carriers, we recommend considering both PCR assays, and to classify animals as positives if they tested positive with at least one of the two RT-PCR tests used.

There have been relatively few studies investigating *Leptospira* spp. in urban parks [51, 52], despite these later serving as green spaces where humans spend extended hours outdoors. The potential risk of exposure to a contaminated environment by small mammals in these areas has been reported in some studies [17, 19, 53]. Moreover, the majority of studies have focused on a single host species, in particular brown rats [54], which are considered as the main reservoir of *Leptospira* spp. in urban area, rather than examining the whole community of small mammals. The levels of *Leptospira* spp. prevalence reported in urban environments may therefore probably reflect these sampling biases towards rats.

Our study emphasized that the levels of *Leptospira* spp. prevalence vary among different small mammal species. Our study showed that the *Leptospira* spp. prevalence was higher in *Rattus norvegicus* (26.1%), especially compared to bank voles and wood mice. This prevalence in rats is comparable to values reported in previous studies conducted in French [11, 19, 25, 26, 55, 56] and other European cities [8, 44, 57]. Our result reinforces the rat’s significance as a key carrier of leptospires. This might be related to their large population size, as well as their life-history traits (longevity, competence), their social behavior, or their presence in sewage [17].

This study is the second to document the circulation of *Leptospira* spp. in small mammals, apart from rats, within European urban environments [58]. Hence, our reference points correspond to *Leptospira* spp. detection in small mammal communities sampled in European forests and meadows [11, 43, 52, 59–62]. From these studies, we expected to detect *Leptospira* in all the small mammal species studied here, with prevalence levels showing large variations and potentially reaching up to 30%. Our findings confirm the circulation of *Leptospira* spp. in four rodent species (*C*. *glareolus*, *M*. *musculus*, *A*. *flavicollis* and *A*. *sylvaticus*) as well as one shrew species (*C*. *russula*) inhabiting urban environments. Lower levels of prevalence were detected in bank voles (5.2%) and wood mice (10.9%) compared to rats, while prevalence did not differ between shrews (9.6%), yellow-necked mice (18.4%) and house mice (6.8%). None of the 29 captured *M*. *arvalis* were tested positive for *Leptospira* spp., a result that may appear surprising with regard to other studies from Germany and Spain, that reported 4.6 to 30.3% prevalence in this vole [43, 62].

These variations in *Leptospira s*pp. prevalence between species and studies might result from specific characteristics of the small mammals analyzed, including their competence to these bacteria, their dispersal, their social and exploratory behaviors. They may also reflect the influence of geographical features on *Leptospira* transmission and maintenance in its reservoirs. Here we have found a higher level of prevalence in the peri-urban park compared to the urban one, while Vitale *et al*. [58] detected higher prevalence in green areas compared to residential areas in Palermo. Geography may shape *Leptospira* spp. distribution in small mammal communities through its impacts on the presence / absence and population size of host species, the composition and diversity of host communities, as well as landscape features (humidity, presence of water points, fragmentation of favorable habitats for small mammal species…). As such, the higher prevalence of *Leptospira* spp. observed in *A*. *sylvaticus* and *C*. *russula*, in the peri-urban compared to the urban park, might be related to some of these geographic features. It would be interesting to deepen our knowledge on the ecology of these species. We specifically recommend conducting research to explore potential ecological, evolutionary and behavioral differences for these host species between habitats with contrasted levels of anthropization and urbanization. This investigation should help understanding how local characteristics influence the epidemiology of *Leptospira* spp. in its host reservoirs.

Temporal surveys are also of great relevance to analyze the impact of abiotic factors on *Leptospira* spp. circulation and maintenance. We describe strong seasonal and year variations in *Leptospira* spp. prevalence. The observed increase between 2020 and 2022 could be an artifact due to differences in sample preservation between these years. It can hence not be interpreted here. We also detected higher prevalence of *Leptospira* spp. in autumn compared to spring, what corroborates previous studies that described the seasonality of leptospirosis in relation to rainfall, ground humidity, and temperature [16, 43, 52, 62, 63]. However, to limit the risk of over-fitting and to favor model parsimony, we did not include any interaction between the different variables included in the model (e.g., between mass and species or site and season). This approach could have masked certain effects specific to a given site or species or, on the contrary, led to the generalization of an effect specific to one species or site to all sites or species. It also limits our conclusion at the global scale of the factors considered (e.g. the whole range of rodent species present, not each individual one), but it is more reasonable than taking the risk of wrongly concluding that there is an effect in a subgroup because of a limited number of data in that subgroup.

The genetic characterization of *Leptospira* infections in small mammals revealed low species diversity and strong bacteria species–host species associations. These results have to be taken cautiously, as molecular data could be collected successfully for only 5.8% of samples with Ct values higher than 37, resulting in a lack of genetic information for low-level positive samples, as previously reported [64]. This could induce biases in the interpretation of the distribution of *Leptospira* spp. genetic diversity with regard to the small mammal communities studied.

*L*. *interrogans* serovar Icterohaemorrhagiae was detected in rats, aligning with the findings of previous studies conducted in Lyon [19] and mainland France [8]. Furthermore, it is the predominant serovar found in humans and dogs in the same region and in the same years in France [24]. This suggests the key role of rats in the transmission risk of leptospirosis. Besides, we detected only one serogroup circulating in the four other rodent species analyzed (*A*. *flavicollis*, *A*. *sylvaticus*, *M*. *musculus* and *C*. *glareolus)*, namely *L*. *interrogans* genotype related to Australis serogroup. These findings partially confirm prior research conducted in European grasslands and forests [61]. The genotype related to Australis was also found in these rodent species. The serogroup Australis is also reported as the second predominant group in dogs in France [24, 65, 66], justifying that the vaccination valances for the dog have been updated with the incorporation of Australis serogroup. The present results underline the potential infectious risk for domestic animals like dogs when frequenting urban and peri-urban parks where rodents are established. Nevertheless, the genetic diversity detected in this study was lower than what has been described in non-urban areas over Europe. Indeed, most of the existing literature on this topic reports the detection of two or three *Leptospira* spp. (*L*. *interrogans*, *L*. *borgpetersenii* and *L*. *kirschneri*). in each of these rodent species [43, 62]. Understanding why *L*. *interrogans* only is found in these urban adapter species, within urban environments [58], is an issue that requires further confirmation and investigation. Similarly, we only detected *L*. *kirschneri* genotype related to the Grippotyphosa serogroup in the shrew *C*. *russula*, while other studies also detected L. *borgpetersenii* and *L*. *interrogans* [52, 62]. Hence, it is worth considering that shrews may also serve as reservoirs for *Leptospira* spp. in urban green areas, and conducting additional surveys to test the potential presence of other *Leptospira* species in these animals is warranted.

Altogether, these findings regarding *Leptospira* characterization in small mammal communities within urban green areas suggest that, despite animals being exposed to the same environments within each park, each small mammal species and individual animal has been infected by a single *Leptospira* strain. This host specificity had been previously described in rats, mice, bank voles, beavers and hedgehogs [10, 19]. But most recent studies found opposite patterns, with several *Leptospira* strains circulating in each host species within small geographical areas. Even intra coinfections were detected using specific techniques and targets [67, 68]. The host specificity with regard to *Leptospira* strain, and how it varies in relation to the landscape, especially with urbanization, requires further investigation.

## 5. Conclusion

Our study shows a significant carriage of leptospires in small terrestrial mammals present in two urban parks in Lyon metropolis, with strong variations observed between animal species and parks. *Leptospira* spp. carriage in small terrestrial mammals seemed to be higher in the peri-urban park and in autumn, potentially due to abiotic factors and to differences in the composition and diversity of small mammal communities. Our study suggested an important public health relevance of rats (*Rattus norvegicus)*, but also of all other small mammal species except the common vole. Commensal and urban adapter rodent species (*Apodemus spp*. and *Clethrionomys glareolus*) seem to be reservoirs of *L*. *interrogans*, while shrews (*Crocidura russula*) seem to serve as *L*. *kirschneri* reservoirs.

Additional research is now critical to gain a deeper understanding of the impact of urbanization and urban greening in European cities on the transmission and maintenance of *Leptospira* within wildlife. Conducting surveys in a broader range of urban and peri-urban parks is needed to obtain more generalized findings and formulate effective preventive measures against leptospirosis risk. These spatial studies also require long-term investigations to assess the influence of environmental factors on *Leptospira* spp. in urban areas, considering climate change, associated extreme weather conditions, and potential changes in the biodiversity of small mammal communities.

## Supporting information

S1 Table List of samples, including sample identification, location (park = site, trapping line, GPS identifications), date of sample collection (day, month and year), season, session, small mammals’ species, sex, mass, length, age, sexual maturation characteristics and RT-PCR results for *Leptospira* spp. and genotyping results (*lfb1* nucleotide sequences). (XLSX)

S2 Table *Leptospira* spp. detection using *lipL32* and 16S rRNA target. (DOCX)

S3 Table Results of the logistic regression model based on the analysis of *lipL32* and 16S rRNA positive animals. (DOCX)

S4 Table Species and serovars detected by genotyping for *Leptospira* DNA extracted from renal tissue. (DOCX)

S1 File Additional supporting information is available from https://doi.org/10.15468/bn8zz7. (DOC)
