# Supplementary material for: Prevalence, genetic diversity and eco-epidemiology of pathogenic Leptospira species in small mammal communities in urban parks Lyon city, France
Source: PLoS One. 2024 Apr 10;19(4):e0300523. doi: 10.1371/journal.pone.0300523 (PMC11006123; doi:10.1371/journal.pone.0300523)
Supplement: S2 Table — (DOCX) [file pone.0300523.s002.docx]

**Table S2.** *Leptospira* spp. detection using *lipL32* and 16S rRNA target.

| **Target gene** | ***lipL32*** | **16S rRNA** | **Total samples in both RT-PCR screening** |
| --- | --- | --- | --- |
| **Number of DNA samples** | 595 | 595 | 595 |
| **Number of positives (n=68)** | 31 | 56 | 19 |
| **Ct ≤ 30** | 15 | 15 | 15 |
| **30 > Ct ≤ 36** | 4 | 11 | 2 |
| **36 > Ct ≤ 40** | 12 | 30 | 2 |
