# Supplementary material for: Prevalence, genetic diversity and eco-epidemiology of pathogenic Leptospira species in small mammal communities in urban parks Lyon city, France
Source: PLoS One. 2024 Apr 10;19(4):e0300523. doi: 10.1371/journal.pone.0300523 (PMC11006123; doi:10.1371/journal.pone.0300523)
Supplement: S3 Table — (DOCX) [file pone.0300523.s003.docx]

**Table S3.** Results of the logistic regression model based on the analysis of *lipL32* and 16S rRNA positive animals, showing the influence of site, year, season, species on the probability of *Leptospira* spp. infection. *Microtus arvalis* was excluded from the statistics analysis in the absence of positive animals. ORs: Odds ratio; 95% IC: 95% confidence interval.

a) 16S rRNA*+lipL32* Analyses (2020-2022)

| **Factor** | **ORs estimated** | **95% IC** | **p value** |
| --- | --- | --- | --- |
| **Location ID: FRPDLL** | 2.21 | [1.04 ;4.66] | 0.0380 |
| **Year: 2021** | 4.81 | [1.81 ;12.74] | 0.0016 |
| **Year: 2022** | 12.45 | [3.47 ;44.67] | 0.0001 |
| **Season: Fall** | 2.24 | [1.16 ;4.31] | 0.0163 |
| ***Rattus norvegicus*** | 3.36 | [1.48 ;7.63] | 0.0038 |
| ***Mus musculus*** | 1.01 | [0.27 ;3.83] | 0.9866 |
| ***Apodemus sylvaticus*** | reference | reference | reference |
| ***Apodemus flavicollis*** | 1.49 | [0.56 ;3.99] | 0.4273 |
| ***Clethrionomys glareolus*** | 0.29 | [0.09 ;0.9] | 0.0327 |
| ***Crocidura russula*** | 1.14 | [0.46 ;2.78] | 0.7802 |

b) 16S rRNA*+lipL32* Analyses (2021-2022)

| **Factor** | **ORs estimated** | **95% IC** | **p value** |
| --- | --- | --- | --- |
| **Location ID: FRPDLL** | 1.66 | [0.76 ;3.64] | 0.2062 |
| **Year: 2022** | 2.24 | [0.95 ;5.29] | 0.0654 |
| **Season: Fall** | 2.08 | [1.07 ;4.04] | 0.0303 |
| ***Rattus norvegicus*** | 3.14 | [1.37 ;7.19] | 0.0070 |
| ***Mus musculus*** | 1.04 | [0.27 ;4.02] | 0.9571 |
| ***Apodemus sylvaticus*** | reference | reference | reference |
| ***Apodemus flavicollis*** | 1.30 | [0.42 ;3.97] | 0.6493 |
| ***Clethrionomys glareolus*** | 0.35 | [0.11 ;1.1] | 0.0724 |
| ***Crocidura russula*** | 1.09 | [0.42 ;2.84] | 0.8546 |
