# Supplementary material for: Prevalence, genetic diversity and eco-epidemiology of pathogenic Leptospira species in small mammal communities in urban parks Lyon city, France
Source: PLoS One. 2024 Apr 10;19(4):e0300523. doi: 10.1371/journal.pone.0300523 (PMC11006123; doi:10.1371/journal.pone.0300523)
Supplement: S4 Table — (DOCX) [file pone.0300523.s004.docx]

**Table S4.** Species and serovars detected by genotyping for *Leptospira* DNA extracted from renal tissue.

*L. interrogans* samples genotyped on the 24 samples submitted to MST and SLST-*lic12008* gene target methods.

|  | **MST profile** | | | | **SLST *lic12008*** | ***L. interrogans* genotyping** |
| --- | --- | --- | --- | --- | --- | --- |
| **Sample** | **MST-1** | **MST-3** | **MST-9** | **Profile** | **Ict./Cop.** | **Serogroup/Serovar** |
| ***Rattus norvegicus* (n=5)** | 4 | - | 3 | Incomplete | Complete  Ict. | Icterohaemorrhagiae Icterohaemorrhagiae |
| ***Rattus norvegicus* (n=2)** | - | - | - | Incomplete | Complete  Ict. | Icterohaemorrhagiae Icterohaemorrhagiae |
| ***Rattus norvegicus* (n=2)** | - | - | - | Incomplete | Incomplete | Icterohaemorrhagiae Icterohaemorrhagiae/Copenhageni |
| ***Mus musculus* (n=1)** | - | - | - | Incomplete | NA | Australis/Autumnalis/Bataviae |
| ***Apodemus sylvaticus* (n=3)** | - | - | - | Incomplete | NA | Australis/Autumnalis/Bataviae |
| ***Apodemus sylvaticus* (n=6)** | 5 | 11 | 6 | Complete | NA | Australis (Jalna/Bratislava/Muenchen) |
| ***Apodemus flavicollis* (n=4)** | 5 | 11 | 6 | Complete | NA | Australis (Jalna/Bratislava/Muenchen) |
| *Clethrionomys glareolus* (n=1) | 5 | 11 | 6 | Complete | NA | Australis (Jalna/Bratislava/Muenchen) |

*L. kirschneri* samples genotyped on the 3 samples submitted to VNTR and O-antigen methods.

|  | **VNTR profile** | | | | **O-Antigen** | ***L. kirschneri* genotyping** |
| --- | --- | --- | --- | --- | --- | --- |
| **Sample** | **VNTR-4** | **VNTR-7** | **VNTR-10** | **Profile** | **GRIP** | **Serogroup/Serovar** |
| ***Crocidura russula* (n=2)** | 0 | 2 | 9 | Complete | Positive | Grippotyphosa Valbuzzi |
| ***Crocidura russula* (n=1)** | 0 | 2 | - | Incomplete | Positive | Grippotyphosa serogroup |
