# Supplementary material for: Prevalence, genetic diversity and eco-epidemiology of pathogenic Leptospira species in small mammal communities in urban parks Lyon city, France
Source: PLoS One. 2024 Apr 10;19(4):e0300523. doi: 10.1371/journal.pone.0300523 (PMC11006123; doi:10.1371/journal.pone.0300523)
Supplement: S1 File — (DOC) [file pone.0300523.s005.doc]

Additional supporting information is available from <https://doi.org/10.15468/bn8zz7>
